# Personalized Federated Learning Based on Dynamic Parameter Fusion and Prototype Alignment

**DOI:** 10.3390/s25165076

**Published:** 2025-08-15

**Authors:** Ying Chen, Jing Wen, Shaoling Liang, Zhaofa Chen, Baohua Huang

**Affiliations:** 1 School of Computer and Electronic Information, Guangxi University, Nanning 530004, China; 2313393009@st.gxu.edu.cn (Y.C.); 2207310118@st.gxu.edu.cn (Z.C.); 2 Guangxi Key Laboratory of Digital Infrastructure, Guangxi Zhuang Autonomous Region Information Center, Nanning 530000, China; wenjing@gxi.gov.cn (J.W.); liangsl@gxi.gov.cn (S.L.)

**Keywords:** federated learning, Non-IID data, dynamic parameter fusion, prototype alignment

## Abstract

To address the limitation of generalization of federated learning under non-independent and identically distributed (Non-IID) data, we propose FedDFPA, a personalized federated learning framework that integrates dynamic parameter fusion and prototype alignment. We design a class-wise dynamic parameter fusion mechanism that adaptively fuses global and local classifier parameters at the class level. It enables each client to preserve its reliable local knowledge while selectively incorporating beneficial global information for personalized classification. We introduce a prototype alignment mechanism based on both global and historical information. By aligning current local features with global prototypes and historical local prototypes, it improves cross-client semantic consistency and enhances the stability of local features. To evaluate the effectiveness of FedDFPA, we conduct extensive experiments on various Non-IID settings and client participation rates. Compared to the average performance of state-of-the-art algorithms, FedDFPA improves the average test accuracy by 3.59% and 4.71% under practical and pathological heterogeneous settings, respectively. These results confirm the effectiveness of our dual-mechanism design in achieving a better balance between personalization and collaboration in federated learning.

## 1. Introduction

With the enactment of data protection regulations such as the General Data Protection Regulation (GDPR), concerns over data privacy have grown significantly in recent years. As multiple data holders cannot share data directly, data silos have become increasingly aggravated. Federated Learning (FL) [1] has emerged as a promising solution to mitigate the challenges associated with such data fragmentation. FL is a distributed machine learning paradigm that enables collaborative model training across multiple institutions without compromising data privacy. Specifically, each client performs local model updates and transmits the updated parameters to a central server for aggregation, rather than sharing raw data [2]. This decentralized approach preserves privacy by ensuring that data remains on local devices, thereby safeguarding user information while effectively harnessing the value of distributed data sources. FL has been widely adopted across various domains, including healthcare, finance, smart cities, and the Internet of Things (IoT). In particular, it has shown strong potential in edge computing and distributed sensing environments, such as smartphones, wearable devices, industrial IoT systems, and smart homes. These sensors continuously generate privacy-sensitive data that are distributed across many devices. The privacy-preserving and communication-efficient nature of FL makes it particularly well-suited for sensor-based applications, enabling model training directly on sensor data without the need for data centralization.

Despite its privacy-preserving benefits, FL faces significant challenges in real-world deployments, particularly those arising from data heterogeneity. In real-world scenarios, clients often differ substantially in label distributions, feature distributions, and sample sizes, leading to non-independent and identically distributed (Non-IID) data characteristics [3]. In such scenarios, the directions and magnitudes of gradient updates from different clients can differ markedly, posing challenges for the aggregated global model to achieve effective generalization across all clients. To address the challenges, personalized federated learning (PFL) techniques have emerged and can be broadly categorized into two paradigms: global model personalization and learning personalized models [4]. The former focuses on training a strong global model and then adapting it to individual clients. For example, FedProx [5] introduces a regularization term in the loss function to constrain local updates, preventing client models from deviating excessively from the global model. SCAFFOLD [6] maintains a control variate for each client to adjust the direction of local updates, thereby aligning them more closely with the global optimization objective. However, these methods often assume that a shared global model can be effectively adapted to diverse local data. In practice, this adaptation is often suboptimal when client data distributions are highly heterogeneous, leading to degraded performance and limited personalization. To overcome these limitations, the second paradigm—learning personalized models—has attracted increasing attention. This approach trains a distinct model for each client, aiming to adapt models to the unique data distributions of individual clients.

Existing PFL methods that follow the paradigm of learning personalized models are mainly based on knowledge transfer, parameter fusion, and parameter decoupling [7,8]. Knowledge transfer-based methods convey global knowledge through soft labels or intermediate features. Representative algorithms include FedHKD [9] and FedProto [10]. However, these methods often overlook the value of preserving local knowledge continuity, making it difficult to maintain stable semantic representations across rounds. Parameter fusion-based methods employ static or adaptive weights to blend parameters from local and global models. Representative algorithms include APFL [11], FedFomo [12], FedAMP [13], and APPLE [14]. While these methods are efficient, they typically perform parameter fusion at the whole-model level. This coarse fusion strategy makes them less effective in scenarios with severe label skew. Parameter decoupling-based methods, such as FedVF [15], FedBABU [16], and FedCAC [17], divide models into shared and personalized modules to achieve an FLexible balance between personalization and generalization. Nonetheless, these methods often neglect the coordination between the two parts, limiting the collaborative effect between shared and personalized modules and ultimately hindering overall model performance.

To address these limitations, we follow the paradigm of learning personalized models and propose a novel PFL framework that integrates the core ideas of knowledge transfer, parameter fusion, and parameter decoupling. Specifically, the model is partitioned into a feature extractor and a classifier. Two mechanisms are introduced: a class-wise dynamic parameter fusion mechanism and a prototype alignment mechanism based on global and historical information. The class-wise dynamic parameter fusion mechanism fuses parameters at the class level, rather than applying a uniform fusion strategy to the entire model. Compared to conventional coarse-grained fusion approaches, this fine-grained strategy offers enhanced control and adaptability under label-skewed scenarios. The mechanism processes each class individually and applies distinct fusion strategies to different class-specific parameters. It adaptively adjusts the fusion ratio between local and global classifier parameters based on the prediction performance of each class. This enables the model to retain reliable client-specific knowledge while effectively incorporating beneficial global information. Meanwhile, the prototype alignment mechanism aligns local features with the global prototypes and the historical prototypes. This alignment strategy enhances semantic consistency across clients while maintaining the stability of local representations. Compared to methods that rely solely on global knowledge, our approach effectively mitigates performance degradation caused by unstable global semantics in scenarios with low participation rates or high data heterogeneity.

The main contributions of this paper are as follows:

- We propose a class-wise dynamic parameter fusion mechanism that achieves fine-grained fusion of classifier parameters. This mechanism adaptively weights and fuses local and global classifier parameters based on class prediction performance, enhancing both personalization and generalization capabilities of the local model.
- We introduce a prototype alignment mechanism based on global and historical information, which jointly constrains local features through global and historical alignment. This effectively mitigates cross-client semantic shifts and enhances the stability of local feature spaces.
- We conduct extensive experiments under various typical Non-IID scenarios, demonstrating that the proposed method surpasses baseline algorithms in both personalization performance and convergence stability.

## 2. Related Work

### 2.1. Knowledge Transfer-Based Personalized Federated Learning

Knowledge transfer-based methods transfer model knowledge between clients or between clients and the server through techniques such as knowledge distillation and prototype alignment. FedKD [18] adopts an adaptive bidirectional knowledge distillation mechanism. Each client trains a large teacher model locally while collaboratively learning a lightweight student model through federated training. Bidirectional knowledge distillation is performed between the two models using soft labels and feature representations. pFedSD [19] addresses the issue of personalized knowledge forgetting by introducing a self-distillation mechanism. Each client retains its historical personalized model and guides the local training process using the Kullback–Leibler (KL) divergence, thereby preserving personalized information across communication rounds. FedHKD [9] introduces the concept of “hyper-knowledge,” which comprises class-mean representations and soft predictions, and shares this hyper-knowledge among clients for knowledge transfer. FedProto [10] exchanges class prototypes, which are defined as the mean feature representations of samples within the same class, between clients and the server. It enforces the alignment of local and global prototypes through a regularization constraint. FedNH [20] addresses the class imbalance problem by initializing class prototypes uniformly on a hypersphere and incorporating class semantic information to enhance the quality of feature representations.

While these methods primarily focus on capturing global knowledge, they often overlook the importance of preserving local knowledge continuity across communication rounds. In contrast, our prototype alignment mechanism effectively incorporates both global and historical local knowledge, thereby enhancing semantic consistency and ensuring representation stability, which is particularly beneficial in scenarios with low client participation or high data heterogeneity.

### 2.2. Parameter Fusion-Based Personalized Federated Learning

Parameter fusion-based methods facilitate personalized modeling through the adaptive fusion of model parameters from diverse sources. APFL [11] realizes personalization by dynamically fusing the global and local models, adjusting the fusion ratio adaptively during training based on local data. FedFomo [12] selects and fuses model parameters from other clients based on relevance, utilizing a weighted combination of performance and parameter distance to personalize the model. FedAMP [13] employs an attention mechanism for parameter aggregation. Each client computes attention weights based on model similarity and aggregates models from similar clients, thereby promoting collaboration among clients with similar data distributions. APPLE [14] introduces learnable Directed Relationship (DR) vectors to adaptively fuse models from other clients, allowing each client to prioritize models from those with similar data distributions. pFedLA [21] adopts a layer-wise parameter aggregation strategy, computing hierarchical fusion weights through a client-specific hypernetwork to capture inter-client similarities at different model depths. FedALA [22] proposes an adaptive local aggregation module that learns element-wise aggregation weights, enabling selective integration of global model parameters that are beneficial to the local objective.

While most existing methods perform parameter fusion at the model level, FedALA [22] introduces a finer-grained approach to selectively integrate global parameters aligned with local objectives. Building on this insight, we propose a class-wise parameter fusion strategy that operates at the class level. This enables each client to retain reliable local knowledge while selectively incorporating beneficial global information. Such class-specific fusion enables more precise personalization and performs better under severe label skew, where coarse-grained methods often struggle to adapt.

### 2.3. Parameter Decoupling-Based Personalized Federated Learning

Parameter decoupling-based methods typically partition model parameters into shared and personalized components to enable more flexible optimization strategies. FedVF [15] divides the model into global and personalized layers, frequently updating the shallow (global) layers to learn general features, while infrequently updating the deep (personalized) layers to enhance local adaptation. FedRep [23] employs a shared global feature extractor and a personalized local classifier; clients perform multiple local updates on the classifier while collaboratively updating the shared representation. AlignFed [24] decouples the model into a personalized feature extractor and a shared global classifier. Feature alignment is achieved by encouraging features to move closer to global class centers, enabling the shared classifier to aggregate knowledge within the aligned feature space. Fed-ROD [25] divides the model into a generic predictor and a personalized predictor, optimizing the former via Balanced Risk Minimization (BRM) and the latter via Empirical Risk Minimization (ERM). FedBABU [16] employs a decoupled training strategy, where the classifier remains fixed throughout the federated training phase. Only the feature extractor is aggregated across clients. In the personalization phase, each client fine-tunes its classifier locally. FedCAC [17] dynamically identifies critical parameters and adopts differentiated sharing strategies. In the early training stages, critical parameters are shared broadly across most clients. As training progresses, sharing becomes restricted to clients with similar data distributions. Meanwhile, non-critical parameters remain globally shared throughout to preserve collaborative benefits.

However, some existing decoupling-based methods treat the shared and personalized components independently, lacking effective coordination between them. This limits the collaborative potential of the two parts and may hinder overall model performance. In contrast, our method enhances the interaction between the feature extractor and the classifier through two complementary mechanisms: class-wise dynamic parameter fusion and prototype alignment. This coordinated design enables more effective personalization while preserving global consistency.

## 3. Preliminaries

### 3.1. Non-IID Data in Federated Learning

In FL, each client often holds a locally collected dataset that differs significantly in distribution due to factors such as variations in data acquisition environments, user behaviors, or task-specific contexts. Such differences result in the Non-IID problem, posing a fundamental challenge to the effectiveness of FL systems. Under Non-IID conditions, each client performs local training based on its specific data distribution, leading to considerable discrepancies in the direction and magnitude of model updates. These inconsistencies hinder the convergence of the global model and degrade its generalization performance across clients. In some cases, the global model may perform worse on individual clients than models trained exclusively on local data, reducing clients’ motivation to participate in collaborative training.

Non-IID data has been broadly classified into several categories, including label skew, feature skew, quantity skew, and concept skew. Label skew refers to the scenario where the label distributions vary significantly across clients, with some clients possessing only a subset of classes, or even a single class. Feature skew denotes the situation where data samples from different clients exhibit significant differences in their feature distributions. Quantity skew refers to the scenario where clients hold varying amounts of data, leading to uneven contributions during training. Concept skew denotes the phenomenon where data samples sharing the same class label across different clients exhibit different underlying feature–label relationships or semantic meanings.

Label skew is the most common and representative type of heterogeneity. For instance, in a human activity recognition scenario using wearable sensors, one client may primarily record walking and sitting activities, while another captures mostly running and jumping, resulting in skewed label distributions across clients. This discrepancy poses significant challenges for training a global model that generalizes well for all participants. In this work, we focus on the label skew problem, as it is one of the primary factors leading to performance degradation in FL and is prevalent in real-world scenarios. To tackle this issue, we propose a novel PFL framework that integrates dynamic parameter fusion and prototype alignment.

### 3.2. Federated Learning Framework and Objective

A typical FL system comprises a central server and N distributed clients. In each communication round, the server randomly selects a subset S of clients to participate in training, where $\left|S\right|=M=\left\lfloor \rho \cdot N\right\rfloor$, and ρ denotes the client participation rate. The server broadcasts the global model parameters ω to the selected clients. Each client i∈S updates its model parameters $\omega_{i}$ with its private dataset $D_{i}$. The parameter update rule for $\omega_{i}$ is given by $\omega_{i}\leftarrow \omega -\eta \nabla L\left(\omega ,x_{k},y_{k}\right)$, where η denotes the learning rate, and $L\left(\omega ,x_{k},y_{k}\right)$ is the loss computed on a sample $\left(x_{k},y_{k}\right)∊D_{i}$. After local updates, each client transmits its updated parameters $\omega_{i}$ to the server. The server then aggregates the received parameters via weighted averaging to update the global model: $\omega \leftarrow \sum_{i=0}^{M-1}\frac{\left|D_{i}\right|}{\left|D\right|}\omega_{i}$, where $\left|D_{i}\right|$ is the number of local training samples on client i, and $\left|D\right|=\sum_{i=0}^{N-1}\left|D_{i}\right|$.

The objective of FL is to minimize the average loss of the global model parameters ω over the data of all participating clients:

(1) $$\underset{\omega }{\min}\sum_{i=0}^{M-1}\frac{\left|D_{i}\right|}{\left|D\right|}L_{i}\left(\omega ;D_{i}\right),$$

where $L_{i}\left(\omega ;D_{i}\right)$ denotes the loss of the global model on dataset $D_{i}$.

### 3.3. Problem Definition

We consider an FL scenario where each client i maintains local model parameters $\omega_{i}$. We assume the local model $\omega_{i}$ consists of two components: feature extractor parameters $\phi_{i}$ and classifier parameters $\theta_{i}$. Formally, $\omega_{i}=\phi_{i}\circ \theta_{i}$, where ∘ denotes model composition. The feature extractor maps the local dataset $D_{i}$ from the input space to the feature space, denoted as $\phi_{i}\left(D_{i}\right)$. The classifier takes the extracted features $\phi_{i}\left(D_{i}\right)$ as inputs and maps them to the output space, expressed as $\theta_{i}\left(\phi_{i}\left(D_{i}\right)\right)$. The objective is to minimize the sum of local losses across all participating clients:

(2) $$\underset{\omega_{0},\ldots ,\omega_{M-1}}{\min}\sum_{i=0}^{M-1}L_{i}\left(\omega_{i};D_{i}\right)=\sum_{i=0}^{M-1}L_{i}\left(\phi_{i}\circ \theta_{i};D_{i}\right),$$

where $L_{i}\left(\omega_{i};D_{i}\right)$ denotes the local loss of client i computed on its dataset $D_{i}$.

## 4. The Proposed FedDFPA Approach

### 4.1. Class-Wise Dynamic Parameter Fusion

**Figure 1:**
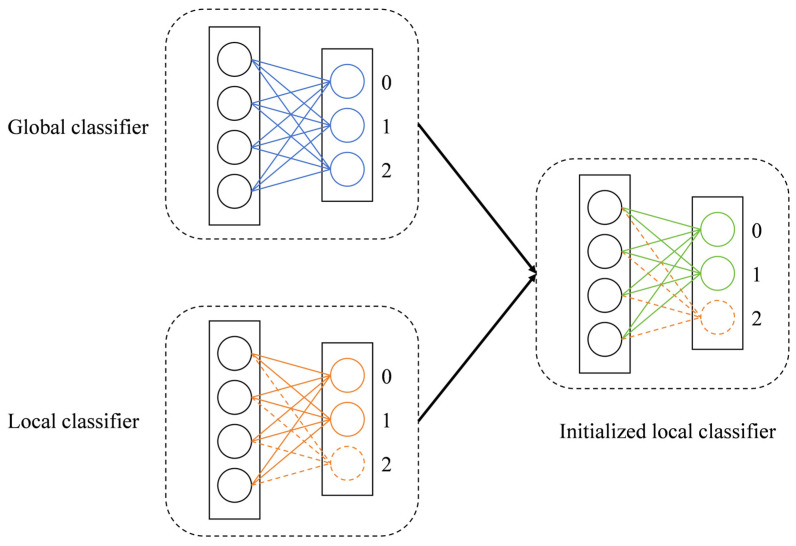
Illustration of the proposed class-wise dynamic parameter fusion mechanism on a representative client with partial class coverage.

Figure 1 illustrates the proposed class-wise dynamic parameter fusion mechanism, taking a representative client as an example. As shown in Figure 1, each neuron in the final layer of the classifier corresponds to a specific class, and its connections to all neurons in the previous layer represent the parameters associated with that class. Assume the local dataset of this client includes samples from class 0 and class 1, but none from class 2. Upon receiving the global classifier parameters, the client dynamically fuses the parameters associated with the classes present in its local dataset. Specifically, the parameters associated with class 0 in both the global and local classifiers are fused using a dynamic coefficient, yielding a new initialization for that class. The same procedure is applied to class 1. For the absent class 2, the corresponding parameters from the local classifier are preserved without modification.

To formally describe this process, we provide a detailed explanation that combines model architecture with mathematical formulations. Specifically, the model is decoupled into a feature extractor $\phi_{i}$ and a classifier $\theta_{i}$. The classifier parameters are denoted as $\theta_{i}\in R^{d_{h}\times \left|C\right|}$, where $d_{h}$ is the hidden layer dimension and $\left|C\right|$ is the number of classes in the global class set C. At initialization, the server independently initializes the global model parameters $\left(\phi_{g},\theta_{g}\right)$, and each client i independently initializes its local parameters $\left(\phi_{i},\theta_{i}\right)$. Each client identifies its local class set $C_{i}\subseteq C$, consisting of the labels present in its local dataset $D_{i}$. Due to the common occurrence of label skew in FL, the local class set $C_{i}$ is typically a subset of the global class set C, and can vary significantly across clients.

In each communication round t, the server broadcasts the global classifier parameters $\theta_{g}^{t-1}$ to all clients. Upon receiving these global parameters, each client performs class-wise dynamic parameter fusion to selectively integrate global knowledge with its local model in a class-wise manner. For each class c in the global class set C, client i first verifies whether the class exists in its local dataset. If $c\in C_{i}$, the client evaluates the performance of its local classifier on class c by computing the classification accuracy ${acc}_{i}^{c}$ on the validation set. Similarly, it computes the classification accuracy ${acc}_{g}^{c}$ of the global classifier on the same class. These two values indicate the relative performance of the local and global classifiers for class c. To balance these two sources of information, we introduce an adaptive fusion coefficient $\alpha_{c}$, defined as follows:

(3) $$\alpha_{c}=\frac{1}{1+exp\left(-\frac{{acc}_{i}^{c}\;-\;{acc}_{g}^{c}}{{acc}_{i}^{c}\;+\;{acc}_{g}^{c}\;+\;\epsilon }\right)},$$

where ϵ is a small positive constant (e.g., $10^{-8}$) to prevent division by zero. This formula dynamically adjusts the fusion weight according to the accuracy difference between the local and global classifiers. When the local classifier outperforms on class c, $\alpha_{c}$ nears 1 to prioritize local parameters. Otherwise, if the global classifier is superior, $\alpha_{c}$ approaches 0, emphasizing global knowledge. Using this adaptive coefficient, the classifier parameter for class c is updated through a combination:

(4) $${\hat{\theta }}_{i}^{t,c}\leftarrow \alpha_{c}\cdot \theta_{i}^{t-1,c}+\left(1-\alpha_{c}\right)\cdot \theta_{g}^{t-1,c},$$

For classes absent locally $\left(c\notin C_{i}\right)$, the client skips the fusion process. Instead, for these classes, the client retains the parameters from the previous round without modification: ${\hat{\theta }}_{i}^{t,c}\leftarrow \theta_{i}^{t-1,c}$. This prevents the incorporation of potentially misleading global information for unseen classes, thereby avoiding negative transfer and preserving the reliability of the local classifier. An initialized local classifier is derived after processing the parameters corresponding to each class. Then, each client performs training based on the initialized local classifier. After local training is completed, each client uploads the updated classifier parameters $\theta_{i}^{t}$ to the server. The server then aggregates the classifier parameters to obtain the new global classifier $\theta_{g}^{t}$, which will be broadcast in the next communication round.

In summary, the proposed class-wise dynamic parameter fusion mechanism contributes to balancing local adaptability and global generalization at the class level. It enhances model robustness by preserving reliable local information while selectively leveraging beneficial global insights. The method selectively integrates global classifier parameters only for classes present locally. This prevents blindly applying mismatched global knowledge, which could degrade performance under Non-IID data.

### 4.2. Prototype Alignment Based on Global and Historical Information

We propose a prototype alignment mechanism based on global and historical information. It leverages global and historical prototypes to ensure global semantic consistency and enhances the stability of feature representations on each client. Our prototype alignment mechanism comprises two parts: distance-based alignment and direction-based alignment. The distance-based alignment minimizes the mean squared error (MSE) between the feature representation of each sample and the corresponding global prototypes of the same class. It ensures that shared class representations among clients are spatially close in the feature space. The direction-based alignment treats the feature representation of each sample and its corresponding historical prototype of the same class as a positive pair. It encourages their cosine similarity to be maximized in contrastive learning—a representation learning approach that pulls similar (positive) pairs together while pushing dissimilar (negative) pairs apart in the embedding space [26]. This direction-based alignment aims to stabilize the local feature representations over time.

Specifically, in each communication round t, the server broadcasts the global classifier parameters $\theta_{g}^{t-1}$ and the global prototypes ${\left\{P_{c}^{t-1}\right\}}_{c\in C}$ to all clients. After dynamic parameter fusion, each client computes class prototypes based on its local data. For each class $c\in C_{i}$, the prototype is defined as the mean of the feature embeddings of all samples belonging to that class, i.e.,

(5) $$P_{i,c}^{t}=\frac{1}{\left|D_{i,c}\right|}\sum_{\left(x,y\right)\in D_{i,c}}\phi_{i}\left(x\right),$$

where $D_{i,c}$ denotes the set of training samples on client i belonging to class c. During local training, each client retains the local prototypes from the current round as historical prototypes for the next round.

Then, the client computes the distance-based alignment loss by measuring the discrepancy between the feature embedding and its corresponding global prototype. Simultaneously, the direction-based alignment loss is computed based on the angular difference between the feature embedding and its corresponding historical prototypes. The combination of these two components constitutes the overall alignment loss $L_{align}$. It is defined as follows:

(6) $$L_{align}=\frac{1}{\left|B\right|}\sum_{\left(x,y\right)\in B}\left[{\left\|\phi_{i}\left(x\right)-P_{y}^{t-1}\right\|}_{2}^{2}+0.5\times \left(1-cos\left(\phi_{i}\left(x\right),P_{i,y}^{t-1}\right)\right)\right],$$

where B denotes a mini-batch of training samples, and $P_{y}^{t-1}$ and $P_{i,y}^{t-1}$ represent the global prototype and the historical local prototype of class y, respectively.

The objective function for local model optimization integrates the cross-entropy loss $L_{CE}$ and the alignment loss $L_{align}$. The total loss is formulated as follows:

(7) $$L\left(\phi_{i}^{t-1}\circ {\hat{\theta }}_{i}^{t};D_{i}\right)=L_{CE}+L_{align},$$

After each training round, the client uploads the updated local prototype ${\left\{P_{i,c}^{t}\right\}}_{c\in C_{i}}$ to the server. The server collects these prototypes and updates the global prototypes for each shared class c by averaging the corresponding local prototypes:

(8) $$P_{c}^{t}=\frac{1}{\left|S_{t,c}\right|}\sum_{i\in S_{t,c}}P_{i,c}^{t},$$

where $S_{t,c}$ is the set of clients containing class c in round t. The server then redistributes the updated global prototypes ${\left\{P_{c}^{t}\right\}}_{c\in C}$ to the relevant clients for the next training round.

In this mechanism, MSE is employed to align local features with their corresponding global prototypes. As a widely used geometric metric, MSE directly measures the distance between two vectors in the feature space, enabling precise alignment. Since global prototypes are typically aggregated from class-specific prototypes across multiple clients, they serve as shared semantic centers representing each class. Therefore, aligning local features to global prototypes using MSE facilitates semantic consistency across clients and alleviates feature shift caused by data heterogeneity. To further enhance the stability of local representations, we compute the contrastive loss between the current local features and their historical prototypes. Unlike MSE, contrastive loss emphasizes the alignment of semantic directions by maximizing the cosine similarity between the current features and historical prototypes.

In summary, the proposed strategy not only promotes semantic consistency across clients for shared classes but also provides more stable representations for local models. By relying on accumulated knowledge from global and historical prototypes rather than the immediate participation of all clients, this mechanism allows the model to maintain robust performance even under fluctuating client participation rates. Therefore, it provides strong adaptability to varying participation scenarios in FL.

### 4.3. The Process of FedDFPA

**Figure 2:**
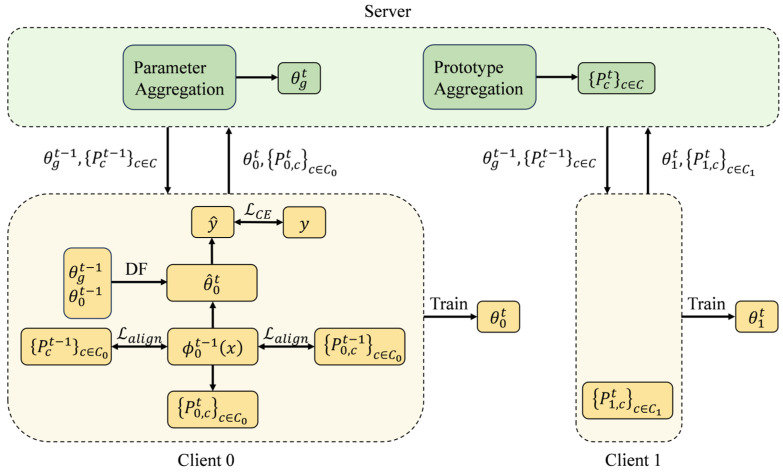
Overview of the FedDFPA framework.

An overview of the FedDFPA framework is illustrated in Figure 2. The complete workflow consists of the following four steps.

Step 1: The server first distributes the global classifier parameters and global class prototypes to all clients.

Step 2: Each client initializes its local classifier using the dynamic parameter fusion mechanism based on the received global classifier parameters.

Step 3: Each client computes local class prototypes for the current round. It then updates local model parameters by optimizing a composite objective function that incorporates both classification loss and prototype alignment loss. Afterwards, the client uploads its local prototypes and the updated classifier parameters to the server.

Step 4: The server aggregates the classifier parameters and class prototypes uploaded by clients to generate the updated global classifier and global class prototypes, completing one communication round. This process is repeated iteratively until convergence.

The details of FedDFPA are presented in Algorithm 1.
**Algorithm 1.** FedDFPA**Input:** N, Total clients; ρ, Client sampling ratio per round; T, Total communication rounds; C, Global category set; $\epsilon =10^{-8}$, Small positive constant; η, Learning rate of local models**Output:** personalized local models $\left[\omega_{0},\omega_{1},\ldots ,\omega_{i},\ldots ,\omega_{N-1}\right]$
**Initialization:** Server initializes global classifier $\theta_{g}^{0}$ randomly, and empty global prototype set ${\left\{P_{c}^{0}\right\}}_{c\in C}$, each client i initializes local model $\omega_{i}^{0}=\phi_{i}^{0}\circ \theta_{i}^{0}$, identifies local class set $C_{i}$ from dataset $D_{i}$
1: for communication round t=1 to T do2:         Server:3:                 Sample client subset $S_{t}$ with ratio ρ
4:                 Send $\theta_{g}^{t-1}$ and ${\left\{P_{c}^{t-1}\right\}}_{c\in C}$ to clients in $S_{t}$
5:         Client ${i\in S}_{t}$ (Parallel):6:                 for each class c∈C:7:                         if $c\in C_{i}$:8:                                Calculate local accuracy ${acc}_{i}^{c}$ and global accuracy ${acc}_{g}^{c}$
9:                                Calculate fusion coefficient $\alpha_{c}$ by $\alpha_{c}=\frac{1}{1+exp\left(-\frac{{acc}_{i}^{c}\;-\;{acc}_{g}^{c}}{{acc}_{i}^{c}\;+\;{acc}_{g}^{c}\;+\;\epsilon }\right)}$
10:                             Update classifier: ${\hat{\theta }}_{i}^{t,c}\leftarrow \alpha_{c}\cdot \theta_{i}^{t-1,c}+\left(1-\alpha_{c}\right)\cdot \theta_{g}^{t-1,c}$
11:                       if ${c\notin C}_{i}$, retain ${\hat{\theta }}_{i}^{t,c}\leftarrow \theta_{i}^{t-1,c}$
12:               end for13:               Calculate local prototypes ${\left\{P_{i,c}^{t}\right\}}_{{c\in C}_{i}}$
14:               Calculate cross-entropy loss $L_{CE}$ with ${\phi_{i}^{t-1},\hat{\theta }}_{i}^{t}$
15:               Calculate prototype alignment loss $L_{align}$
16:               Update local model: ${\phi_{i}^{t},\theta }_{i}^{t}\leftarrow {\phi_{i}^{t-1},\hat{\theta }}_{i}^{t}-\eta \nabla \left(L_{CE}+L_{align}\right)$
17:               Upload $\theta_{i}^{t}$ and ${\left\{P_{i,c}^{t}\right\}}_{{c\in C}_{i}}$ to server18:       Server:19:               Update global classifier: $\theta_{g}^{t}=\frac{1}{\left|S_{t}\right|}\sum_{i\in S_{t}}\theta_{i,c}^{t}$
20:               Update global prototypes ${\left\{P_{c}^{t}\right\}}_{c\in C}$ for each class c∈C: $P_{c}^{t}=\frac{1}{\left|S_{t,c}\right|}\sum_{i\in S_{t,c}}P_{i,c}^{t}$
21: end for22: return personalized local models $\left[\omega_{0},\omega_{1},\ldots ,\omega_{i},\ldots ,\omega_{N-1}\right]$


## 5. Experiments

### 5.1. Datasets and Non-IID Settings

We conduct experiments on two widely used image classification datasets, CIFAR-10 and CIFAR-100 [27], as well as the text classification dataset AG News [28]. To simulate the Non-IID data settings commonly encountered in FL, we adopt two partitioning strategies:

- Practical Non-IID Setting [29]: The data is partitioned using a Dirichlet distribution Dir (β), where the parameter β controls the degree of data heterogeneity. A smaller β results in more skewed and imbalanced label distributions across clients. We set the value of β to 0.1.
- Pathological Non-IID Setting [1,30]: Each client is assigned data samples from a fixed number of classes—2 classes per client for CIFAR-10 and 10 classes per client for CIFAR-100.

For both settings, the local dataset of each client is further split into training, validation, and testing sets with a ratio of 8:1:1. The distribution of the validation set and test set is consistent with that of the local training set. This setup allows evaluation of the model’s local generalization performance under Non-IID conditions.

### 5.2. Training Details

In the experiments, we employ a lightweight four-layer Convolutional Neural Network (CNN) as the backbone model for image datasets and use the fastText model for the text dataset. The CNN begins with two convolutional layers for feature extraction. The first convolutional layer uses 32 filters with a kernel size of 5 × 5, followed by a ReLU activation and a 2 × 2 max pooling operation. The second convolutional layer employs 64 filters and similarly applies ReLU activation and 2 × 2 max pooling. Given the input image size of 32 × 32 × 3, as in CIFAR-10 and CIFAR-100, the output of the final convolutional layer is a feature map of size 5 × 5 × 64, which is then flattened into a 1600-dimensional vector. This feature vector is fed into two fully connected layers. The first fully connected layer projects the input to 512 hidden units, followed by a ReLU activation. The second layer serves as the classifier head, producing a 10-dimensional output for CIFAR-10 and a 100-dimensional output for CIFAR-100.

We simulate a federated environment with 20 clients. For the image classification tasks, the total number of communication rounds is set to 200 to ensure model convergence; for the text classification task, it is set to 100. Each client performs local training using Stochastic Gradient Descent (SGD) with a learning rate of 0.01 for image classification and 0.001 for text classification. The batch size is 64, and each client runs two local epochs per round for image tasks and one local epoch per round for the text task.

Our development environment applies Python (version 3.9) and PyTorch (version 1.12.1) with hardware acceleration of a single NVIDIA GeForce RTX 3060 GPU (NVIDIA Corporation, Santa Clara, CA, USA).

### 5.3. Results and Discussion

To evaluate the effectiveness of our proposed method FedDFPA, we conduct experiments on the CIFAR-10 and CIFAR-100 datasets under two types of data heterogeneity: practical heterogeneity and pathological heterogeneity. We compare it against five state-of-the-art PFL baselines: APPLE [14], FedALA [22], FedKD [18], FedProto [10], and FedCAC [17]. We run all the experiments three times and report the mean and standard deviation.

Table 1 reports the average test accuracies of all local models achieved by FedDFPA and other algorithms on the CIFAR-10 and CIFAR-100 datasets under practical heterogeneous setting. Table 2 presents the results under pathological heterogeneous setting. As shown in Table 1, on the CIFAR-10 dataset, FedDFPA consistently outperforms all baseline methods across both participation rates. Specifically, it reaches accuracies of 91.08% and 91.19% under client participation rates of 0.5 and 1.0, respectively, surpassing the strongest competitor FedALA by margins of 0.89% and 0.72%. On the more challenging CIFAR-100 dataset, where the label space is larger and class imbalance is more severe, FedDFPA maintains its superiority with 54.64% and 55.45% accuracy. It achieves up to 1.09% gain over FedALA. Notably, methods such as APPLE and FedProto show significant drops in performance under lower participation, indicating their limited robustness in highly heterogeneous and partially participating environments. In contrast, FedDFPA maintains strong performance. When the participation rate decreases from 1.0 to 0.5, FedDFPA shows only a 0.81% drop in accuracy, which is significantly smaller than APPLE (12.46%) and FedProto (4.71%). This indicates the strong robustness of FedDFPA to varying participation rates.

As shown in Table 2, under the pathological heterogeneous setting of CIFAR-10, FedDFPA achieves 91.29% and 91.56%. It consistently outperforms FedALA and FedCAC by approximately 0.8–1.3%, with larger margins over APPLE and FedKD. On CIFAR-100, FedDFPA continues to deliver superior results. It achieves 66.36% accuracy at 0.5 participation and 67.24% at 1.0, outperforming the next-best method, FedKD, by 0.53% and 1.18%, respectively. Some methods suffer significant performance degradation when the participation rate drops. For example, APPLE experiences a 5.63% drop and FedProto a 7.06% drop. In contrast, FedDFPA shows only a 0.88% reduction, demonstrating remarkable stability and robustness to varying participation levels. Overall, these results confirm the effectiveness of FedDFPA. The substantial improvements underscore its strong capability to mitigate the challenges of data heterogeneity in FL.

On CIFAR-100, we observe that the accuracy under Dirichlet partitioning is lower than that under the pathological partition. This is mainly due to the difference in how class distributions are assigned to each client. In pathological partitioning, each client receives data from a fixed and limited set of classes (e.g., 10 for CIFAR-100), which enables the local model to better adapt to its local distribution. In contrast, even with a low concentration parameter (e.g., β=0.1), Dirichlet partitioning allocates a larger number of classes to each client compared to pathological partitioning but allocates fewer samples per class. High class diversity combined with limited samples per class increases the difficulty of local optimization. This effect is more pronounced on CIFAR-100 due to its larger number of classes, which require more data per class for effective learning.

**Figure 3:**
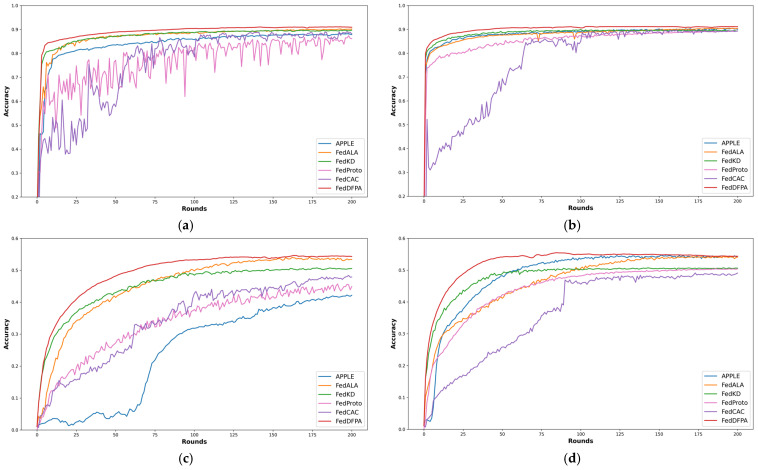
Average accuracy curves on CIFAR-10 and CIFAR-100 in the practical heterogeneous setting. (**a**) Average accuracy curves on CIFAR-10 with a client participation rate of 0.5; (**b**) average accuracy curves on CIFAR-10 with a client participation rate of 1.0; (**c**) average accuracy curves on CIFAR-100 with a client participation rate of 0.5; (**d**) average accuracy curves on CIFAR-100 with a client participation rate of 1.0.

**Figure 4:**
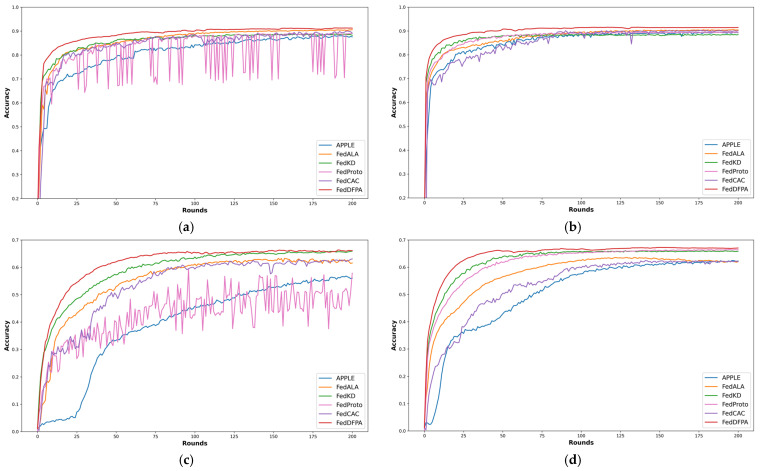
Average accuracy curves on CIFAR-10 and CIFAR-100 in the pathological heterogeneous setting. (**a**) Average accuracy curves on CIFAR-10 with a client participation rate of 0.5; (**b**) average accuracy curves on CIFAR-10 with a client participation rate of 1.0; (**c**) average accuracy curves on CIFAR-100 with a client participation rate of 0.5; (**d**) average accuracy curves on CIFAR-100 with a client participation rate of 1.0.

Figure 3 and Figure 4 illustrate the test accuracy curves of various methods throughout the training process, enabling a direct comparison of their convergence behavior and stability under different levels of data heterogeneity. Specifically, Figure 3 presents the performance of FedDFPA and baseline methods on CIFAR-10 and CIFAR-100 in the practical heterogeneous setting. Figure 4 shows the results under the pathological heterogeneous scenario.

In Figure 3a (client participation rate = 0.5), FedDFPA and three other PFL methods reach relatively high accuracy early in the training process, significantly outperforming FedProto and FedCAC. When considered alongside Figure 3b (client participation rate = 1.0), it becomes evident that FedProto and FedCAC are particularly sensitive to client participation rates. Both methods suffer from large accuracy fluctuations and reduced convergence stability at lower participation rates. This instability can be attributed to the design of FedProto, which aligns local prototypes solely with the current global prototypes, rather than incorporating alignment with historical prototypes as in FedDFPA. FedProto lacks mechanism to stabilize local feature representations over time. Due to lower client participation, the global prototype undergoes substantial shifts across rounds, causing frequent changes in the optimization direction of local models. For FedCAC, low participation leads to insufficient coverage of similar clients, disrupting collaboration among key model parameters and further destabilizing performance. Figure 3c,d show the results on the CIFAR-100 dataset. The APPLE method also exhibits notable sensitivity to changes in client participation rates. When the participation rate decreases from 1.0 (Figure 3d) to 0.5 (Figure 3c), APPLE’s performance drops substantially, falling from the second-best to the worst among all methods. This is because, under highly heterogeneous and low-participation conditions, the number of available core models is significantly reduced, resulting in the loss of some class-specific knowledge. Although APPLE uses Directed Relationship (DR) vectors to weight other clients’ core models, when some class-specific models are missing, the Directed Relationship (DR) vector cannot fully compensate, leading to poor generalization on those classes.

As observed in Figure 4, FedDFPA consistently achieves the highest final accuracy and faster convergence. Moreover, it exhibits minimal performance fluctuation throughout training, indicating strong stability. In most scenarios, FedKD and FedALA emerge as the strongest competitors, both demonstrating competitive accuracy and robust training stability. This can be attributed to their thoughtful design: FedKD benefits from its communication-efficient mentor–mentee framework and adaptive knowledge distillation, which allows clients to retain rich local representations while collaboratively training a compact model. FedALA effectively mitigates statistical heterogeneity through its Adaptive Local Aggregation (ALA) module. It learns fine-grained, element-wise aggregation weights to selectively fuse beneficial global knowledge into the local model, thereby enhancing personalization. However, FedKD’s performance is limited by its reliance on sufficient local data to train effective mentor models. FedALA lacks the mechanism to align global and local prototypes during training, which can hinder convergence in extremely heterogeneous environments. In contrast, FedDFPA integrates dynamic parameter fusion and prototype alignment to better capture both shared and personalized knowledge, leading to superior performance across diverse data distributions and client participation rates.

**Figure 5:**
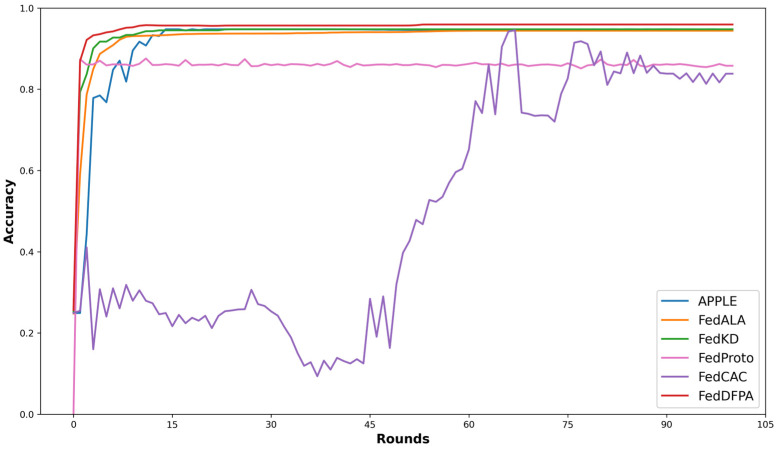
Average accuracy curves on AG News in the practical heterogeneous setting with a client participation rate of 1.0.

To further evaluate the generalizability of FedDFPA, we additionally conducted experiments on the AG News dataset in the practical heterogeneous setting with a client participation rate of 1.0. As shown in Figure 5, FedDFPA consistently achieves higher test accuracy across communication rounds, demonstrating its effectiveness in text-based FL scenarios.

In summary, FedDFPA demonstrates strong convergence stability and final test performance under different levels of data heterogeneity and client participation rates. These advantages are primarily attributed to the proposed dynamic parameter fusion and prototype alignment mechanisms, which not only facilitate efficient optimization but also enhance the model’s adaptability to local data distributions.

### 5.4. Communication Cost Analysis

To evaluate the communication efficiency of FedDFPA, we compare its communication overhead with five PFL methods. The comparison focuses on the type and volume of data exchanged between the client and the server in each communication round. As shown in Table 3, APPLE requires each client to upload only its local model but necessitates downloading multiple models from other clients, resulting in substantial downstream communication overhead that scales with the number of clients. In FedALA, the client and the server exchange the entire model parameters each round. FedKD reduces communication cost by exchanging only the compressed gradients of the mentee model, using dynamic compression based on singular value decomposition (SVD). In FedProto, each client uploads its local class prototypes and downloads the aggregated global prototypes. Since prototypes are significantly smaller than full models, this approach substantially reduces communication overhead. FedCAC requires each client to upload its local model along with a binary mask indicating sensitive parameters. The server then distributes both a global model and a customized global model to each client, thereby increasing both upload and download communication overhead. In FedDFPA, each client uploads its local class prototypes and classifier parameters and downloads the aggregated global class prototypes and global classifier. Since class prototypes are low-dimensional embeddings and the classifier parameters correspond only to the final layer of the model, the total communication volume is significantly smaller than that of full model exchange. Therefore, FedDFPA incurs lower communication overhead compared to methods such as APPLE, FedALA, and FedCAC.

In addition to communication overhead, it is also important to consider the computational cost of the method. Compared with FedAvg [1], FedDFPA introduces moderate computation overhead. Specifically, each client computes the local and global classifier accuracies to determine fusion weights and further calculates the MSE and the contrastive loss. Despite these additional computations, the increase in computational overhead does not significantly affect training efficiency. These operations contribute to FedDFPA’s improved performance and faster convergence.

### 5.5. Ablation Study

To validate the effectiveness of the two core components in FedDFPA, namely dynamic parameter fusion and prototype alignment, we conduct an ablation study. We evaluate the individual and joint contributions of these components to the overall model performance. Experiments were conducted on the CIFAR-10 and CIFAR-100 datasets under the practical Non-IID setting. We evaluated the performance under two different client participation rates: 0.5 and 1.0. The results are summarized in Table 4. We denote the version with only dynamic parameter fusion as FedDF, and the version employing solely prototype alignment as FedPA. As shown in Table 4, both modules individually contribute to performance improvements, while their combined application consistently yields the best results across all settings. For instance, on CIFAR-10 with a participation rate of 1.0, the complete FedDFPA achieves 91.19% accuracy, surpassing its variants FedDF and FedPA by 0.75% and 0.24%, respectively.

These results confirm that both components of FedDFPA make independent and complementary contributions to performance enhancement. The dynamic parameter fusion mechanism focuses on personalized modeling by adaptively adjusting the fusion strategy according to inter-class variation, better preserving local information. In contrast, the prototype alignment mechanism promotes consistency in globally shared semantic representations, thereby improving the generalization ability of the model. When used together, the two mechanisms achieve a balance between personalization and shared knowledge, leading to superior overall performance across various scenarios.

**Figure 6:**
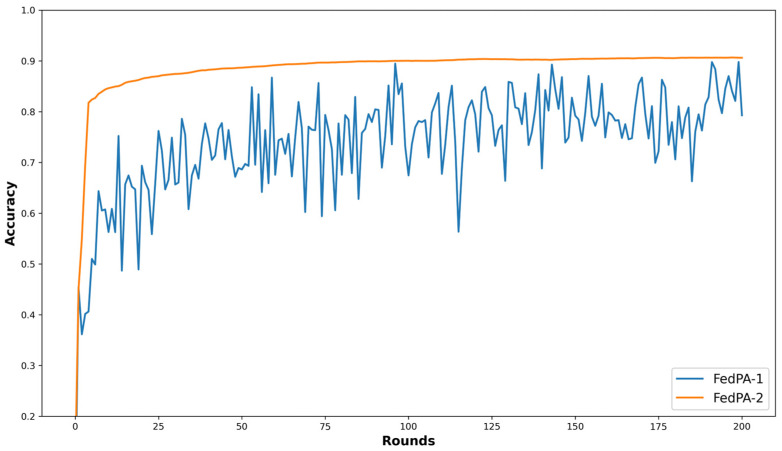
Average accuracy curves of FedPA-1 and FedPA-2 on CIFAR-10 in the practical heterogeneous setting with a client participation rate of 0.5.

To further investigate the effectiveness of incorporating historical prototypes, we conducted an ablation study comparing two variants of FedPA: FedPA-1, which uses only the global prototypes, and FedPA-2, which leverages both global and historical prototypes. The average test accuracy curves of these two variants on the CIFAR-10 dataset with a client participation rate of 0.5 are presented in Figure 6. As shown in Figure 6, FedPA-1 exhibits noticeable oscillations in accuracy across communication rounds, indicating unstable learning. In contrast, FedPA-2 shows a much smoother and more stable learning curve, demonstrating that the use of historical prototypes helps to reduce fluctuations during training. This can be attributed to the fact that, under data heterogeneity and low client participation, global prototypes often shift across communication rounds. Relying solely on these shifting global prototypes for alignment introduces variability in local model updates, making the training process more unstable. These observations suggest that incorporating historical prototypes can effectively mitigate semantic drift and stabilize model updates in heterogeneous FL environments.

## 6. Limitations and Future Work

Although FedDFPA demonstrates strong performance under various Non-IID scenarios, several limitations remain to be addressed in future work. First, compared to standard FL methods, FedDFPA introduces higher computational overhead at the client side. Second, while the prototype alignment mechanism enhances training stability using historical prototypes, its memory overhead grows linearly with the number of clients and classes, which may pose scalability challenges in large-scale deployments. Future research could explore prototype compression techniques to improve memory efficiency. Third, FedDFPA relies on the assumption that local labels are both available and accurate enough to enable class-wise accuracy computation. However, this assumption significantly limits its applicability in real-world deployments. In many practical scenarios, clients operate under weak supervision, where only a portion of the data is labeled. Additionally, label noise is common in user-annotated datasets, making it difficult to obtain accurate class-specific performance metrics. In such cases, the resulting classifier fusion ratios may become unreliable, ultimately degrading overall model performance. Future work could explore different strategies to address this limitation. For weak supervision, pseudo-labeling techniques that generate soft or confident labels during training may help approximate class-wise performance without requiring fully labeled data. For noisy label scenarios, incorporating label noise detection and confidence-weighted fusion can allow the model to adaptively downweight unreliable information, thereby improving the robustness of classifier fusion.

In summary, future work could focus on improving the efficiency, scalability, and robustness of FedDFPA in more practical and challenging FL environments. This includes reducing client-side computation and memory overhead, developing more compact prototype representations, and designing adaptive mechanisms to handle incomplete or noisy supervision. By addressing these limitations, FedDFPA could be extended to broader real-world applications where data heterogeneity, label quality, and resource constraints are major concerns.

## 7. Conclusions

In this paper, we address the limited generalization capability of traditional FL under Non-IID data by proposing a novel PFL method, FedDFPA. This method integrates two core mechanisms: a class-wise dynamic parameter fusion mechanism and a prototype alignment mechanism based on both global and historical information. The former adaptively fuses local and global classifier parameters at the class level, enabling personalized modeling for each class. This allows clients to retain their effective local knowledge while selectively incorporating beneficial information from the global model, thereby enhancing both personalization and generalization capabilities. The latter enhances semantic consistency across the FL system and improves the stability of local feature representations by introducing both distance-based and direction-based alignment. It also effectively alleviates semantic drift caused by data heterogeneity.

We conduct extensive experiments under various typical Non-IID data settings and different client participation rates. The results demonstrate that FedDFPA outperforms five state-of-the-art personalization methods, with accuracy gains ranging from 1.24% to 6.42% under practical settings, and from 1.87% to 4.71% under pathological settings. These findings confirm the effectiveness and robustness of our dual-mechanism design in enhancing personalized modeling and cross-client collaboration.

## Figures and Tables

**Table 1:** Mean test accuracy (%) and standard deviation in the practical heterogeneous setting with different participation rates (ρ=0.5 and ρ=1.0).

Algorithm	CIFAR-10		CIFAR-100	
	ρ=0.5	ρ=1.0	ρ=0.5	ρ=1.0
APPLE	88.20 ± 0.19	90.42 ± 0.19	42.24 ± 0.25	54.70 ± 0.21
FedALA	90.19 ± 0.11	90.47 ± 0.09	54.06 ± 0.10	54.36 ± 0.10
FedKD	89.73 ± 0.13	89.91 ± 0.11	50.74 ± 0.15	50.71 ± 0.13
FedProto	87.36 ± 0.52	89.24 ± 0.55	45.71 ± 0.51	50.42 ± 0.50
FedCAC	89.16 ± 0.54	89.70 ± 0.51	48.37 ± 0.57	49.10 ± 0.58
FedDFPA	91.08 ± 0.16	91.19 ± 0.15	54.64 ± 0.19	55.45 ± 0.18

**Table 2:** Mean test accuracy (%) and standard deviation in the pathological heterogeneous setting with different participation rates (ρ=0.5 and ρ=1.0).

Algorithm	CIFAR-10		CIFAR-100	
	ρ=0.5	ρ=1.0	ρ=0.5	ρ=1.0
APPLE	88.08 ± 0.21	89.49 ± 0.20	56.80 ± 0.26	62.43 ± 0.22
FedALA	90.48 ± 0.09	90.59 ± 0.09	63.25 ± 0.10	63.58 ± 0.09
FedKD	88.75 ± 0.11	88.58 ± 0.11	65.83 ± 0.11	66.06 ± 0.12
FedProto	89.03 ± 0.53	89.51 ± 0.50	59.47 ± 0.51	66.53 ± 0.51
FedCAC	90.03 ± 0.55	90.27 ± 0.54	62.92 ± 0.57	62.43 ± 0.54
FedDFPA	91.29 ± 0.14	91.56 ± 0.14	66.36 ± 0.16	67.24 ± 0.17

**Table 3:** Communication overhead of different algorithms.

Algorithm	Upload	Download
APPLE	Local model	Multiple other client models
FedALA	Local model	Global model
FedKD	Compressed Mentee gradients	Compressed Mentee gradients
FedProto	Local class prototypes	Global class prototypes
FedCAC	Local model and binary mask	Global model and customized global model
FedDFPA	Local prototypes and classifier	Global prototypes and classifier

**Table 4:** Mean test accuracy (%) of FedDFPA vs. other versions on CIFAR-10 and CIFAR-100 with different participation rates (ρ=0.5 and ρ=1.0).

Algorithm	CIFAR-10		CIFAR-100	
	ρ=0.5	ρ=1.0	ρ=0.5	ρ=1.0
FedDF	90.25 ± 0.21	90.44 ± 0.18	54.19 ± 0.22	54.28 ± 0.20
FedPA	90.62 ± 0.19	90.95 ± 0.18	54.40 ± 0.21	55.01 ± 0.21
FedDFPA	91.08 ± 0.16	91.19 ± 0.15	54.64 ± 0.19	55.45 ± 0.18

## Data Availability

The CIFAR-10 and CIFAR-100 datasets are publicly available at: http://www.cs.toronto.edu/~kriz/cifar.html (accessed on 6 June 2025). The AG News dataset is publicly available at: https://pytorch.org/text/stable/datasets.html#ag-news (accessed on 26 July 2025).
